# Draft Genome Sequence of *Mycobacterium tuberculosis* KT-0133, Isolated in South Korea

**DOI:** 10.1128/genomeA.01731-15

**Published:** 2016-02-11

**Authors:** Taesoo Kwon, Seung Jung Han, Won Gi Yoo, Mi-ran Yun, Sanghyun Lee, Jong Seok Lee, Dae-Won Kim

**Affiliations:** a School of Biological Sciences, Seoul National University, Seoul, Republic of Korea; b Division of Biosafety Evaluation and Control, Korea National Institute of Health, Korea Centers for Disease Control and Prevention, Chungbuk, Republic of Korea; c Department of Environmental Medical Biology, Chung-Ang University, Seoul, Republic of Korea; d Department of Microbiology and Institute for Immunology and Immunological Diseases, Brain Korea 21 Plus Project for the Medical Sciences, Yonsei University College of Medicine, Seoul, Republic of Korea; e International Tuberculosis Research Center, Changwon, Republic of Korea

## Abstract

Here, we present the draft genome sequence of *Mycobacterium tuberculosis* KT-0133, which belongs to the Korean-Beijing family. This sequence will provide a new perspective on the evolution and accommodation of *M. tuberculosis* KT-0133 in human hosts.

## GENOME ANNOUNCEMENT

Tuberculosis (TB) remains a public health problem in South Korea, and its causative agent, *Mycobacterium tuberculosis*, infects about one-third of the world’s population. According to a World Health Organization survey, there were approximately 8.8 million new cases of TB causing nearly 1.1 million deaths in 2010 (1). Among the TB strains, the Beijing lineage is a cause of major concern worldwide (2, 3), owing to its global spread, involvement in several TB outbreaks, and association with rapid disease progression. Despite its importance, genome information and reports on Beijing TB are relatively sparse.

*M. tuberculosis* KT-0133 was identified as a member of the Korean-Beijing family MTB according to spoligotyping, and was sensitive to first-line anti-TB drugs. Strain KT-0133 was isolated from the sputum of a retreatment TB patient at Masan National Hospital in South Korea. *M. tuberculosis* KT-0133 was grown in 7H9 broth (Difco Laboratories, USA) supplemented with 10% (vol/vol) oleic acid-albumin-dextrose-catalase (OADC; Becton, Dickinson, USA) for 1 month at 37°C, and the genomic DNA was isolated as previously described.

Using the Nextera sample preparation kit (Illumina, USA), a paired-end sequencing library was constructed for the Illumina MiSeq platform, and the insert size was 500 bp. A total of 4,655,846 reads were produced from the whole-genome sequencing with 259.66-fold coverage. The reads were assembled into 119 contigs with the CLC Genomics Workbench version 7.5 program (CLCbio, USA) (4), and the *N*_50_ size was 99,151. After the assembly, we estimated a genome size of 4,365,676 bp with a 65.6% G + C content for the KT-0133 strain. Using Glimmer version 3.02 (5), 4,114 putative open reading frames (ORFs) were identified. With respect to RNA genes, 45 tRNAs and 3 rRNAs were identified with tRNAScan-SE (6) and RNAMMER (7), respectively. Among the 4,114 ORFs, 2,788 genes could be assigned to COG functional categories; 249 (8.93%) genes were classified to lipid transport and metabolism-related genes, which was the third-ranked category for abundance. This high abundance is likely due to the fact that *M. tuberculosis* has more than 100 outer membrane proteins, and lipids are required to construct the outer membrane (8). Moreover, 2,852 single nucleotide variants (SNVs) and 254 indels were identified with reference to the *M. tuberculosis* H37Rv genome (accession no. NC_000962) with GATK version 3.2.2 (9). Among the SNVs, 78.7% (2,244) were from ORFs, with the rest in intergenic regions. Of the identified indels, 59.5% (91/153) of the insertions and 67.3% (68/101) of the deletions were in ORFs, with the rest identified to intergenic regions.

This genome will provide a new perspective on the evolution and accommodation of *M. tuberculosis* KT-0133 in human hosts.

### Nucleotide sequence accession number.

This whole-genome shotgun project has been deposited in the DDBJ/EMBL/GenBank under the accession number JUFG00000000.
